# Complementary medicine in psychology practice: an analysis of Australian psychology guidelines and a comparison with other psychology associations from English speaking countries

**DOI:** 10.1186/s12906-022-03620-2

**Published:** 2022-06-25

**Authors:** Carrie Thomson-Casey, Jon Adams, Erica McIntyre

**Affiliations:** 1 School of Public Health, Faculty of Health, University of Technology Sydney, Sydney, Australia; 2 Institute for Sustainable Futures, University of Technology Sydney, Sydney, Australia

**Keywords:** Clinical practice, Complementary medicine, Ethics, Policy and guidelines, Integrative mental health

## Abstract

**Background:**

Psychologists, and their clients, are engaging with complementary medicine (CM). Increasing evidence for CM approaches, such as improved nutrition and St John’s wort, has led to their inclusion in the Royal Australian New Zealand College of Psychiatrists clinical practice guidelines for mood disorders. This research aims to determine in what ways, and to what extent, Australian psychology regulatory bodies and associations consider CM relevant to psychology practice. Specifically, how these regulatory bodies and professional association’s ethical and practice guidelines engage with CM.

**Methods:**

Documents from Australian regulatory bodies and professional associations, that relate to the governance or guidance of psychologists’ clinical practice, were systematically searched for key terms relating to CM.

**Results:**

There were no direct references to CM in the 58 ethical and practice guidelines reviewed. There was also no reference to the relevance of CM to ethnocultural groups, such as Aboriginal and Torres Strait Islander traditional healing practices.

**Conclusion:**

While other mental health care disciplines are working toward integrating CM, the discipline of psychology in Australia is not currently engaged in such developments. Given the exponential rise of CM use amongst those with mental health problems, psychology associations should consider developing resources and guidelines to assist psychologists in navigating CM in relation to clinical practice to help minimise risks, such as patient safety associated with concurrent CM use.

## Key Points:

What is already known about this topic.

1. Consumers of mental health services utilise complementary medicine at high rates.
2. Psychologists are already engaging with complementary medicine.
3. Psychologists engaging with complementary medicine are doing so without guidance.

What this topic adds.

1. Current ethical/clinical practice guidelines do not include reference to complementary medicine or traditional healing practices.
2. Current clinical practice guidelines do not meet the current demand from psychologists and their clients.
3. The lack of guidelines puts psychologists and their clients at risk.

Internationally psychologists are engaging with their client’s complementary medicine (CM) use, in some form, with limited policy, clinical practice guidelines, or formal support from their professional associations or regulatory bodies [1, 2]. Studies suggest Australia’s psychology professional associations also do not support psychologists who engage with CM [1, 3–5]. The current article critically evaluates existing guidelines for psychologists with regards to the relationship between CM and psychology.

CM includes a broad range of health care products, services and practices (including traditional medicine practice), that are not part of conventional medicine and “are not fully integrated into the dominant health care system” [6]. CM is often self-selected, including over the counter vitamin and mineral supplements, herbal medicines, traditional medicines, yoga, aromatherapy, meditation and massage [7–11]. There is demand for CM in mental health settings with prevalence rates reaching 82% in some countries [12–15]. People with mental health problems in Australia also have high CM use and it is likely that psychologists will encounter clients who are using at least one form of CM [16].

Internationally the lack of CM-focused guidelines for psychologists has been noted. For example, Siegel and Turato [17] reported that Brazil’s professional association for psychologists has not adequately responded to their National Policy for Integrative and Complementary Practices stating “no specific material on [applied psychology] and CAM [complementary and alternative medicine] has been produced so far” ([6] p. 1529). Similarly, Barimah and Akotia [18] observed that “Despite this growth in consumer demands for complementary medicine or TRM [traditional medicine], the policy responses of African and other governments and health professions have been either absent or inadequate” ([18] p. 100). The discipline of psychology may not be adequately informing psychologists on how they might engage with their client’s preference for CM as part of mental health treatment [3, 19]. In the context of informed consent, Liem’s research on psychologists in Indonesia and Australia [2] reported that participants believed “clients have the right to know all the possible treatments available, including CAM [CM] treatments, and their safety and effectiveness” (p. 6).

Research has demonstrated the efficacy of CM approaches, such as yoga, to address mental health symptoms such as stress, anxiety and low mood [20–22]. There is also strong evidence for ingestible CM, such as the herbal medicines St John’s wort and Saffron in treating mild to moderate depression [23–26]. Nutritional interventions (e.g., Mediterranean diet, probiotics) have also gained empirical support for the prevention and treatment of depression [27–30]. There is evidence to suggest some CM treatments may have a role in helping to address mental health problems [31, 32]. However, there are a number of identified risks associated with the use of some CMs, especially within the context of wider concurrent use alongside pharmacological treatments.

In some countries (e.g., America and England) specific CM approaches are accepted by psychologists and integrated into their practice, such as meditation and mindfulness; yet they were not previously considered a component of psychology [31, 33, 34]. Some psychologists report engaging with CM based on holistic and client centred principles [3, 35, 36], while others discuss client demand and acknowledge the cultural relevance of some CM (Barimah and Akotia 2015 [18, 37–39]. Psychologists are also seeking training in, and already using, some CM approaches in their practice [1, 3, 4, 19]. Prevalence rates for psychologists integrating CM into their practice vary dependent on research aims, methodologies, and what is included in definitions of CM. Up to 83% of psychologists have recommended some form of CM to their clients, 52% had made a referral to a CM practitioner and 65% were directly applying CM as part of their practice [2, 40–42]. For example, a recent study reported more than 50% of Australian mental health practitioners (73.1% of participants were psychologists) recommend improved nutrition and dietary changes for depression, anxiety, and stress, at least weekly in their practice [43]. Similarly, psychologists acknowledge the value of physical activity (e.g., yoga) as part of mental health care treatment and recommend physical activity or refer to movement/exercise-based health professionals [44–46]. Despite consumer and psychologist interest in CM, there is uncertainty among Australian psychologists how to safely integrate these approaches safely into their clinical practice [5, 47].

Australian psychologists note the absence of CM relevant guidelines [1, 48]. The concern for lack of guidelines is reported by participant psychologists in relevant papers, and includes: 1) confusion around the ethical responsibility to advise clients appropriately about CM [5, 49], 2) what is allowable in terms of integrating CM in clinical practice [19, 36], 3) how the efficacy of CM can be explored by psychologists in safe and ethical ways [4, 35], and 4) that psychologists in clinical practice are attempting to address consumer demand for CM without clear policy and guidelines [2, 3, 47]. Australian studies also cite lack of knowledge or education as a barrier to psychologist engagement with CM  [43, 50]. For example, Baxter and Lovell state “mainstream psychological practices to date have not included the use of dietary change or supplementation as part of their training”; however, psychologists in the study, report utilising CM (in this case nutrition) with their clients at least weekly and believe “the role of dietary change for positive mental health has further informed and justified mental health practitioners’ prescription of dietary change” ([50] p. 521). A lack of guidance on CM for psychologists has implications for broader client care, including risks to patient safety, disconnection from emerging research and evidence for mental health treatments that include CM, and psychologists’ potential disconnect from certain clients and ethnocultural groups who may prefer CM treatment options. Further, the absence of CM in guidelines for psychologists may subsequently exclude relevant CM from psychologist tertiary education curricula.

There are several psychology organisations responsible for the development and enforcement of regulations, code of ethics, and clinical practice guidelines for psychologists in Australia, including the Australian Health Professionals Regulation Agency (AHPRA), the Psychology Board of Australia (PsyBA), the Australian Psychology Accreditation Council (APAC) and the Australian Psychological Society (APS). The APS is the largest psychology professional association in Australia with 25,000 members (Australian Psychological Society, n.d.). There are smaller psychology professional associations, such as the Australian Association of Psychologists Incorporated (AAPI) and Australian Clinical Psychology Association (ACPA). Speciality psychology groups beyond the above organisations represent other diverse areas of psychology practice such as the Australian Indigenous Psychologists Association (AIPA), the Australian Psychologists and Counsellors in Schools (APACS) and the Australian Music and Psychology Society (AMPS). There are also psychology speciality groups within the APS such as the College of Health Psychologists and the Psychology and Integrative Mental Health Interest Group. These organisations and groups have varying influence on how psychologists’ practice in clinical settings. Psychologists are guided by their education and once in practice they must adhere to AHPRA guidelines and the APS Code of Ethics and ethical guidelines. The APS also provides “ethical considerations” documents that provide examples of interpretation of the ethical guidelines.

Currently there is no explicit policy and/or guidelines for CM within psychology practice from Australia’s psychology’s professional associations and regulatory bodies. The exception is the APS ethical guideline for hypnosis teaching and use. Hypnosis is often included in definitions of CM [51]. Given the concerns raised in the literature about the lack of guidelines, this article aims to explore how Australian psychology associations and regulatory bodies inform psychologist engagement with CM. This article presents the findings of a document analysis of existing guidelines, for references related to the integration of CM into psychology practice. In addition, a brief comparison of the CM relevant clinical resources provided across Australian, American, British and Canadian psychology associations is explored.

## Method

The READ (Ready materials, Extract data, Analyse data, Distil) approach was applied as a systematic procedure [52] across two phases to analyse documents from Australian and international psychology professional and regulatory bodies. The document analysis aimed to address the research questions; Do Australian psychology associations and regulatory bodies consider CM relevant to psychology practice? How do Australian psychology associations compare to other English speaking countries (The America Psychological Association (APA) and British Psychological Society (BPS)) psychology associations in terms of their engagement with CM topics? Psychology association and regulatory body policy and guidelines were considered credible sources of text that can be analysed using a deductive approach [53]. The first stage of the content analysis involved a superficial review of documents for direct mention of CM within psychology practice. Following the initial review, the decision was made to create a priori codes from the literature to assist data extraction. Key search terms/codes developed were “complementary medicine”, “complementary and alternative medicine”, “complementary therapies”, “integrative medicine”, “integrative health”, “integrative mental health”, “traditional medicine”, and “traditional healing”. Widening the codes to include terms such as nutrition and naturopathy was considered and dismissed to keep the research focused on umbrella terms as representations of CM. Further, a search using an exhaustive list of terms to describe CM professions, products and practices is beyond the scope of the current study.

Phase 1 search was *within* documents produced by Australian psychology professional and regulatory associations around the governance (e.g., registration, auditing, accreditation, complaints and compliance) or guidance of the activities of psychologists in clinical practice (e.g., code of ethics, ethical guidelines). The associations were contacted by email in June 2020 to request information regarding access to their policy and guideline documents. The associations were also asked if they have *policy/guidelines that they have developed (separate to the Australian Psychological Society guidelines) that are relevant to psychologists who might be engaging with CM in their practice.* One of the associations did not respond to an email request for documents (ACPA), and three replied (AIPA, AAPI, APS) by email advising that their association (psychologist members) adheres to the APS code of ethics and ethical guidelines or advised that they are informed by psychology’s regulatory bodies (Psychology Board of Australia and AHPRA). Access to APS policy and guideline documents were available through the first author’s association membership. The remaining documents were retrieved from the Psychology Board (www.psychologyboard.gov.au) and AHPRA (https://www.ahpra.gov.au/). APAC was excluded from the guideline and policy search as they advise psychologist education standards and not clinical practice. A list of included documents is shown in Table 1.

**Table 1:** Frequency of search terms within relevant documents published by the APS, PsyBA and AHPRA

Document type	Title	Organisation	Date	Frequency, n (%)
Code of ethics	APS Code of Ethics	APS	2007	0 (0.0)
Guideline	Guidelines: Mandatory notifications about registered health practitioners	AHPRA	2020	0 (0.0)
Guideline	Guidelines for advertising regulated health services	AHPRA	2020	0 (0.0)
Guideline	Guidelines for supervisors	PsyBA	2018	0 (0.0)
Guideline	Guidelines for supervisor training providers	PsyBA	2018	0 (0.0)
Guideline	Guidelines: Continuing professional development	PsyBA	2015	0 (0.0)
Guideline	Guidelines on area of practice endorsements	PsyBA	2019	0 (0.0)
Guideline	Guidelines for the 4 + 2 internship program	PsyBA	2017	0 (0.0)
Guideline	Guidelines for the National Psychology Exam	PsyBA	2019	0 (0.0)
Guideline	Guidelines for the 5 + 1 internship program	PsyBA	2013	0 (0.0)
Policy	Policy for provisional registration in combined 4th and 5th year programs of study	PsyBA	2019	0 (0.0)
Policy	Common Protocol—Informing notifiers about the reasons for National Board decisions	PsyBA	2018	0 (0.0)
Policy	Policy for recency of practice requirements	PsyBA	2016	0 (0.0)
Policy	Policy on working in addition to placements	PsyBA	2015	0 (0.0)
Policy	Policy for higher degree students applying for general registration	PsyBA	2016	0 (0.0)
Policy direction	Policy Direction 2019–01—Paramountcy of public protection when administering the National Scheme Policy Direction 2019–02—Requirements to consult with patient safety bodies and health care consumer bodies on every new and revised registration standard, code and guidelines	PsyBA	2020	0 (0.0)
Manual	National psychology exam candidate manual	PsyBA	2019	0 (0.0)
Ethical guideline	Ethical guidelines for Aboriginal and Torres Strait Islander Peoples	APS	2015	0 (0.0)
Ethical guideline	Guidelines for the use of therapeutic aversive procedures	APS	2020	0 (0.0)
Ethical guideline	Ethical guidelines on confidentiality	APS	2016	0 (0.0)
Ethical guideline	Ethical guidelines on providing psychological services in response to disasters	APS	2014	0 (0.0)
Ethical guideline	Ethical guidelines regarding financial dealings and fair trading	APS	2020	0 (0.0)
Ethical guideline	Ethical guidelines for psychological practice in forensic contexts	APS	2013	0 (0.0)
Ethical guideline	Ethical guidelines for working with clients when there is a risk of serious harm to others	APS	2013	0 (0.0)
Ethical guideline	Ethical guidelines on the teaching and use of hypnosis	APS	2016	0 (0.0)
Ethical guideline	Ethical guidelines for psychological practice with clients with an intellectual disability	APS	2016	0 (0.0)
Ethical guideline	Ethical guidelines for providing psychological services and products using the internet and telecommunications	APS	2011	0 (0.0)
Ethical guideline	Ethical guidelines for psychological practice with lesbian, gay and bisexual clients	APS	2010	0 (0.0)
Ethical guideline	Ethical guidelines for psychological practice with clients who disclose memories related to traumatic experiences	APS	2018	0 (0.0)
Ethical guideline	Ethical guidelines for psychological practice with men and boys	APS	2017	0 (0.0)
Ethical guideline	Ethical guidelines for psychological services involving multiple clients	APS	2014	0 (0.0)
Ethical guideline	Ethical guidelines for managing professional boundaries and multiple relationships	APS	2016	0 (0.0)
Ethical guideline	Ethical guidelines for working with older adults	APS	2014	0 (0.0)
Ethical guideline	Ethical guidelines relating to procedures that involve psychologist-client physical contact	APS	2016	0 (0.0)
Ethical guideline	Ethical guidelines on providing pro bono or voluntary psychological services	APS	2014	0 (0.0)
Ethical guideline	Ethical guidelines for psychological assessment and the use of psychological tests	APS	2018	0 (0.0)
Ethical guideline	Ethical guidelines on record keeping	APS	2020	0 (0.0)
Ethical guideline	Ethical guidelines on reporting abuse and neglect, and criminal activity	APS	2020	0 (0.0)
Ethical guideline	Ethical guidelines for psychological practice in rural and remote settings	APS	2016	0 (0.0)
Ethical guideline	Ethical guidelines on working with sex and/or gender diverse clients	APS	2013	0 (0.0)
Ethical guideline	Ethical guidelines on the prohibition of sexual activity with clients	APS	2017	0 (0.0)
Ethical guideline	Ethical guidelines relating to clients at risk of suicide	APS	2014	0 (0.0)
Ethical guideline	Ethical guidelines for psychological practice with women and girls	APS	2012	0 (0.0)
Ethical guideline	Ethical guidelines on supervision	APS	2013	0 (0.0)
Ethical guideline	Ethical guidelines for working with and in the media	APS	2018	0 (0.0)
Ethical guideline	Ethical guidelines for working with young people	APS	2018	0 (0.0)
Ethical consideration	Ethical considerations when providing pro bono services	APS	2017^a^	0 (0.0)
Ethical consideration	Ethical considerations when providing second opinions	APS	2017^a^	0 (0.0)
Ethical consideration	Assessing risk of harm to others	APS	2018^a^	0 (0.0)
Ethical consideration	Client suicide: considerations for psychologists	APS	2017^a^	0 (0.0)
Ethical consideration	Contracts and ethical concerns	APS	2017^a^	0 (0.0)
Ethical consideration	Establishing and maintaining boundaries	APS	2017^a^	0 (0.0)
Ethical consideration	Ethical issues in rural practice	APS	2017^a^	0 (0.0)
Ethical consideration	Making sound ethical decisions	APS	2017^a^	0 (0.0)
Ethical consideration	Managing clients at risk of suicide	APS	nd^a^	0 (0.0)
Ethical consideration	Managing multiple relationships	APS	2017^a^	0 (0.0)
Ethical consideration	Psychologist’s clients’ rights	APS	2017^a^	0 (0.0)
Ethical consideration	Record keeping—templates	APS	nd	0 (0.0)

^a^Originally published at an earlier date in InPsych magazine

Included documents were searched (December 2021) using the a priori codes developed from the literature [54]. Search results were extracted and recorded in a spreadsheet with documents as rows and columns recording document details and occurrences of codes. Where a full or partial code (e.g., alternative) appeared in text the whole section was copy and pasted into the spreadsheet with the page number. This allowed interrater checking for relevance and context for each occurrence. Where documents contained key search terms, the surrounding text was examined for contextual relevance to CM in psychology practice. The outcomes measures were the number of relevant occurrences of the code within each document (Table 1).

Phase 2 of the document analysis was a search (December 2021) *across* Australian and comparable English speaking psychology association websites for potentially relevant documents that discuss psychologists in clinical practice engaging with CM. The American Psychological Association (APA), British Psychological Society (BPS), and Canadian Psychology association (CPA) were selected as comparative association websites [55], were in English and were also accessible without association membership. Relevant documents (web pages) were selected from the first page of results returned using search terms. The search terms were a priori codes developed from the literature [54] relating to frequency of CM terms and included “complementary medicine”, “complementary and alternative medicine”, and “complementary therapies”. Returned page headings and descriptions were copy and pasted into an excel spreadsheet and examined for relevance. Relevant occurrences and a sample of documents were recorded (Table 2).

**Table 2:** Search results across Australian and international psychology associations

Association/website	Occurrences n (%)	Sample document type/title/link	Description/context
Australian Psychological Society/psychology.org.au	2/10 (20)	Interest group: Psychology and integrative mental health https://groups.psychology.org.au/PsyCT/	This interest group is concerned with developing and nurturing professional links between psychologists and professional practitioners and researchers working in the field of Complementary and Alternative Medicine (CAM)
Australian Psychological Society/psychology.org.au		Professional development: Complementary medicine: What psychologists need to know https://psychology.org.au/event/21419?view=true	Workshop to cover aspects of complementary medicine within psychology, ethics, evidence, and resources, introduces psychologists to CM products and services broadly
American Psychology Association/ www.apa.org	6/10 (60)	Curriculum: APA Substance Use Disorders Curriculum: For Training Psychology Graduate Students to Assess and Treat Substance Use Disorders https://www.apa.org/ed/graduate/substance-use	Describes intervention approaches including *alternative methods and complementary medicine approaches (acupuncture, *etc*.)*
American Psychology Association/ www.apa.org		Guideline: APA Clinical practice guideline for the Treatment of depression across three age cohorts https://www.apa.org/depression-guideline/guideline.pdf	This guideline addresses the efficacy of psychological and complementary and alternative medicine treatments, the comparative effectiveness of psychotherapy in combination with pharmacotherapy as well as compared to pharmacotherapy and complementary and alternative treatments
American Psychology Association/ www.apa.org		Book: Complementary and Alternative Medicine for Psychologists: An Essential Resource https://www.apa.org/pubs/books/4317345	Provides psychologists with therapists with the information they need to provide advice on the safety and effectiveness of complementary and alternative medicine therapies and describes a broad array of approaches that may benefit clients
American Psychology Association/ www.apa.org		Magazine article: More than psychotherapy http://www.apaservices.org/practice/good-practice/more-than-psychotherapy.pdf?_ga=2.106805830.413613913.1641097362-2012957043.1638914638	Magazine article has discussed psychologists integrating CM into their practice
American Psychology Association/ www.apa.org		Professional development: Alternative techniques https://www.apa.org/monitor/2013/04/ce-corner	“Today’s psychologists are increasingly integrating complementary and alternative medicine techniques into their work with clients. Here’s an overview of the most popular treatments, the research on their efficacy and the ethical concerns they raise.” (APA, n.d.)
British Psychological Society/ https://www.bps.org.uk	6/10 (60)	Interest group: Division of Clinical Psychology Faculty of Holistic Psychology https://www.bps.org.uk/member-microsites/dcp-faculty-holistic-psychology	The Faculty also seeks to apply the accepted models and methodologies of clinical psychology to the psychological aspects of complementary therapies
British Psychological Society/ https://www.bps.org.uk		Briefing paper: Alternatives to antipsychotic medication: Psychological approaches in managing psychological and behavioural distress in people with dementia bps.org.uk/sites/www.bps.org.uk/files/Member%20Networks/Divisions/DCP/Alternatives%20to%20Antipsychotic%20medication.pdf	A form of alternative and complementary medicine based on the use of very concentrated ‘essential’ oils from the flowers, leaves, bark, branches, rind or roots of plants with purported healing properties
British Psychological Society/ https://www.bps.org.uk		Annual conference (Northern Ireland branch) presentation: “A study examining Complementary and alternative medicine (CAM) use and its relationship with Attitudes towards CAM, Holistic Health and Locus of Control of Behaviour, in Adults with Chronic Fatigue Syndrome (CFS) and/or Myalgic Encephalomyelitis (ME) in Ireland.” https://www.bps.org.uk/sites/www.bps.org.uk/files/Member%20Networks/Branches/Northern%20Ireland/2017%20UG%20Session%20Schedule%2023%20March%202017.pdf	Research on Complementary and Alternative Medicine (CAM) use in worldwide populations whilst becoming more frequent, remains linked to more readily recognised diagnoses, such as cancers, musculoskeletal disorders, or arthritis
British Psychological Society/ https://www.bps.org.uk		Presidential blog: Eight months of success in making psychology relevant to citizens and the real world https://www.bps.org.uk/blogs/presidential-blog/eight-months-success-making-psychology-relevant-citizens-and-real-world	Previous (2017) BPS President’s blog “Our position isn’t of course, to reject or oppose diagnosis as an element of care, but to ensure that alternative and complementary approaches are given attention commensurate with their importance.”
British Psychological Society/ https://www.bps.org.uk		Minutes: Minutes of the Meeting of the Covid 19 Coordinating Group (15/07/2020) https://www.bps.org.uk/sites/www.bps.org.uk/files/Policy/Policy%20-%20Files/Covid-19%20Coordinating%20Group%20Meeting%20Minutes%20-%C2%A015%20July%202020.pdf	“The workstream have had discussions around creating guidance relating to providing alternative or complementary outdoor therapies e.g. animal therapy “
Canadian Psychological Association/ https://cpa.ca/		https://cpa.ca/psychology-works-fact-sheet-pediatric-oncology/	Palliative care supports may also address physical, psychological, emotional and social areas of need. It is important to also acknowledge that beyond the aforementioned treatments, a number of additional treatments and supports may also be provided or sought out by families. For example, complementary and alternative medicine approaches such as herbal remedies, diet and nutrition interventions, faith-healing, homeopathy, mind–body therapies, and massage therapy may be used

This study has been reported as per the Standards for Reporting Qualitative Research [56]. To improve the trustworthiness of the findings the authors discussed their biases with each other prior to, and throughout, to ensure that these did not unduly influence the data collection and analysis process. Any disagreements were resolved through author discussion until consensus was reached. No ethical approval was required as all data (web-based documents) were retrieved from the public domain. All methods were performed in accordance with the relevant guidelines and regulations.

## Results

For phase 1, 58 policy and guideline documents were included and searched—42 from the APS, 14 from the Psychology Board, and two from AHPRA—none had a direct reference to the CM search terms. Where key search terms were found in the text they were checked for context with CM. Although several of the search terms were partially present in the text, they were not contextually relevant to CM and therefore not included as an occurrence. For example, the term “alternative” appeared several times; however, it was used in the context of “alternative provider” or “alternative services” and not in the context of complementary and alternative medicine. In the above example “alternative” refers to a different psychologist or a different psychology service. For phase 2 several sample documents (e.g., guidelines, book, professional development activity) were extracted from websites and a description of the occurrences are provided in Table 2. There were less occurrences on the APS (20%) website compared to the APA (60%) and BPS (60%) websites. A search of the Canadian Psychological Association (CPA) was also conducted using the search terms, however the search yielded only one result (10%). The search result did mention CM in the context of psychologists working in paediatric oncology, and encouraged psychologists to be aware that some paediatric oncology clients may also use a variety of CM.

Of note, there was a reference to CM in an APS magazine article available online, which referred to naturopathy—a system of CM practice [57]. The magazine article also appeared as a link to professional resources described as an ethical consideration titled *Managing multiple relationships*. An extract from the question-and-answer style magazine article on managing multiple relationships follows:

“I have qualifications in both psychology and naturopathy and believe my clients would benefit from receiving both services. Can I use both skill sets with my clients?

*Psychologists may be qualified as members of two or more professions. The APS Guidelines for managing professional boundaries and multiple relationships (2008) state that “psychologists can be qualified as members of two or more professions (for example, psychology and law). If a psychologist attempts to work in both roles with the same client there is a strong risk of role confusion for both client and psychologist. Consequently such situations should be avoided.” If psychologists choose to provide two distinct professional services it is advisable that they run two distinct practices servicing different clientele and ideally from different locations.”*

Informal searches also located an AHPRA document *Guidelines for advertising a regulated health service* [58] which contained reference to AHPRA regulated CM professions including traditional Chinese medicine, osteopathy and chiropractic and referred to “herbal” and “acupuncture” in the context of those professions. Similarly, the document *Guidelines on area of practice endorsements* [59] contained reference to “nutrition” in the context of sport and exercise psychology. Similarly, the most recent Australian *Accreditation Standards for Psychology Programs* document [60] was also reviewed for CM relevant content for general interest in the context of this article. Under the heading Master of Sport and Exercise Psychology the document states a competency as "Apply advanced psychological knowledge of … nutrition and eating behaviour … to their practice in sport and exercise”. To the author’s knowledge the current Master of Sport and Exercise Psychology program at an Australian university does not explicitly contain nutrition as a subject. As these documents were not captured using the search terms they are not included in the results tables, however their presence and relevance is discussed below.

## Discussion

The document analysis presented here has established that there is no explicit policy or clinical practice guidelines from Australian regulatory bodies and psychology professional associations to inform psychologist engagement with CM within their clinical practice, despite the growing demand [6, 32, 61] and evidence of efficacy for some forms of CM in mental health treatment [62–64]. This insight is important as it substantiates the concern outlined in previous publications regarding the lack of a clear position, specific directions, or guidelines from Australian psychology associations on their members’ engagement with CM [1, 5].

Although these findings are consistent with international literature that discusses the absence, or inadequacy of, guidelines for psychologists on CM [65, 66] it also highlights that psychology is not engaging with CM as much as other mental health professions such as psychiatry, that do provide guidance. For example, the Royal Australian and New Zealand College of Psychiatrists (RANZCP) clinical practice guidelines for mood disorders recommend some evidence-based CM, such as St John’s wort and nutrition interventions [62]. The National Preventive Health Strategy 2021–2030 [67] also includes nutrition and physical activity as important preventative measures for mental health. Consequently, the omission of CM from psychology’s policy and guidance documents does not align with practices and guidelines of other mental health professions [15, 68].

The finding that there is no direct reference to CM in Australian psychology ethical guidelines is important because it highlights potential safety risks for all psychologists who have clients that use CM. The literature demonstrates that client’s lack of disclosure of their CM use to their health care providers is a safety risk; for example, not disclosing self-prescribed ingestible CM that has potential contraindications with concurrent conventional medicine use [69, 70]. However, even if clients do disclose their CM use, their treating psychologist may lack the knowledge needed to respond appropriately. If psychologists are not informed by their professional associations about how to discuss CM as part of mental health treatment planning there are potential risks, such as being unaware of, and unable to advise clients with regards to possible CM treatment-drug interactions. The inability of psychologists to keep up with demand and/or client preference for CM may partly be due to psychologists’ lack of understanding and/or knowledge of CM [43, 71]. Further, the *National framework for recovery-oriented mental health services* [72] suggests health professionals should have capacity to discuss client’s treatment preferences with the client’s treating practitioners, including CM practitioners. It is important for psychology associations to inform psychologists of how to discuss CM with their clients to ensure client safety.

Psychologists are already referring, recommending, or directly applying CM as part of their client’s treatment planning [4, 41, 43]. Subsequently, the development of guidelines may need to be a priority for Australian psychology associations. With no specific guidelines for CM in psychology and no direct reference to CM in psychology association’s guidelines, psychologists are left to interpret vague guidelines for how they may safely engage with their client’s CM use [5, 73]. There are risks when psychologists are required to interpret indirect and limited clinical guidance for CM, such as breaches to scope of practice (unintentionally or intentionally), inadequate informed consent, and potential for complaints against the psychologist. The literature discusses psychologist’s confusion around interpreting indirect references in policy and guidelines as to what could be considered engagement with CM [2, 66]. Further, there is a lack of clarity on what constitutes sufficient knowledge or competency in CM [1, 4]. The ethical guidelines may be open to interpretation [74, 75] as to how that psychologist would incorporate a formal CM qualification (dual qualifications) into their practice. For example, if a psychologist holds qualifications as both a CM practitioner (e.g., traditional Chinese medicine) and a psychologist, the current “ethical considerations”—on managing multiple relationships—could be considered prohibitive, particularly to those psychologists with advanced knowledge in CM via dual qualifications (discussed below). Psychologists need to interpret such things as contingent liability for referring to a CM practitioner, informed consent when including CM in treatment planning, and potentially managing multiple professional relationships in the context of dual qualifications. Without direct references or adequate guidelines specifically addressing CM in psychology, psychologists engaging with CM could be in breach of their scope of practice and risk complaint to AHPRA or punitive measures from the Psychology Board.

Further, the absence of CM in guidelines for psychologists reflects limitations in psychology’s acknowledgement or engagement with the cultural relevance of CM for some people. For example, the therapeutic relationship may be at risk if a psychologist, unable to engage with their client’s CM use, minimises the cultural relevance of CM for some ethnocultural groups. CM may have greater significance for some ethnocultural groups who use traditional medicines (e.g., Chinese, Indian, Aboriginal and Torres Strait Islander people) seeking support from a psychologist [76–78]. In contrast, although the American Psychological Association’s (APA) Race and Ethnicity Guidelines in Psychology [79], do not directly discuss CM, they aim to address health disparities and recommend psychologists engage with their client’s cultural beliefs [71] and engaging with culturally-oriented practices and ideologies (e.g., traditional Chinese medicine) are invaluable for informing mental health practice [79]. The APA guidelines encourage psychologists to “actively educate themselves about diverse Indigenous/ethnocultural healing modalities” and suggest “training programs could incorporate Indigenous healing theories and practices throughout the curriculum” ([79] p. 25). The APA guidelines also acknowledge that psychology in the “United States and abroad reflect Western European methods and have neglected forms of healing from other ethnocultural perspectives”; further, that psychologists “operating from the dominant Western paradigm often view ‘alternative’ forms of healing, such as ritual healing practices, as inferior to the interventions consistent with the training they received” ([79] p. 24). Although these guidelines encourage psychologists to understand Indigenous and/or ethnocultural resources for healing, there is no direct reference to how these CM approaches could be integrated with psychology practice. The APS does have a document/guideline aimed at addressing the needs of Aboriginal and Torres Strait Islander people; however, this document has no reference to Indigenous traditional healing or Aboriginal and Torres Strait Islander traditional medicine/healing practitioners or practices.

Beyond the absence of CM policy and guidelines, Australian psychology professional associations are not engaging with CM to the same degree as international psychology associations. For example, the APA provide some education and guidance for psychologists interested in CM, and recommendations have been made by APA [80] accreditation agencies that psychologists be “familiar with common medical, dental, and other health treatments, as well as complementary and alternative treatments” ([80] p.16). The APA also encourages engagement with client preference for CM, particularly in the context of patient centred care [71] and “to understand and encourage indigenous/ethnocultural sources of healing within professional practice” ([79] p. 24). Table 2 provides examples of international psychology association’s engagement with CM. The APA and BPS provide resources and professional development opportunities on their website related to CM. In addition, an APA magazine article discussed psychologists integrating CM into their practice including navigating referral to CM practitioners and legal aspects of informed consent when integrating CM into their clinical practice [81]. It is interesting to note that the CPA had limited mention of CM on their website given the Canadian Network for Mood and Anxiety Treatments (CANMAT) Taskforce is inclusive of CM [63]. Similar to the APS, the CPA may not be influenced by medicine and psychiatry’s inclusion of CM in clinical guidelines.

Further, the advice provided in the APS magazine article, regarding dual qualifications in naturopathy, supports the notion that psychology should distance itself from CM [66]. The article does not provide references (other than the APS *Code of Ethics* and *Guidelines for managing professional boundaries and multiple relationships*) that might justify psychology’s distance from CM, or dual health qualifications as unethical for psychologists. References that are provided in the related guideline contrast with the above advice. For example, the referenced APA code of ethics [82] states “multiple relationships that would not reasonably be expected to cause impairment or risk exploitation or harm are not unethical” ([80] p. 1065). Australian psychology association’s disconnection from CM is problematic given the literature and findings from the above-mentioned studies, the integration of some forms of CM into RANZCP guidelines, the APA and BPS resources on CM, the APA flexibility on dual qualifications, and the APA statement on the cultural relevance of CM/traditional healing resources.

Australian psychology associations may consider that CM within psychology is only relevant to a small group of Australian psychologists and that guidelines for CM in psychology practice are not necessary. It is possible that the authors of Australian psychology association guidelines think differently about CM than psychologists in clinical practice [41]. It is also possible that Australian psychology associations are cautious regarding CM in psychology. There are sceptic groups, such as the “Friends of Science” that directly oppose CM in any form and question the legitimacy of CM products and services [83]. The concern of such groups appears to be the potential loss of purity of psychology and risks to evidenced based practice across the sciences, and in some cases these groups also dismiss psychology as a pseudoscience [84, 85]. Further, these sceptic groups are concerned that including CM in tertiary education and guidelines for health professionals “promotes usage rather than a critical rejection of these ineffective and potentially dangerous practices” [86]. There are published commentaries that perceive CM to be a threat to the scientific development and/or scientific standing of psychology [66, 87]. For example, Fasce and Adrian-Ventura [88] state that a psychologist integrating CM into their practice has rejected scientific research and evidence-based practice. Psychologists have also been found to fear negative appraisal from their psychologist peers if they express interest in, or integrate, CM into their clinical practice [2, 48].

The absence of CM relevant guidelines is not commensurate with consumer and psychologist demand for CM within psychology practice, emerging evidence for some CM for some mental health problems, and the cultural relevance of CM. Australian psychology professional associations may need to review and develop contemporary guidelines, that consider the inclusion of CM, and a shift toward an integrative approach. For example, in the context of multiple professional roles and dual qualifications the APA accommodates “Multiple relationships that would not reasonably be expected to cause impairment or risk exploitation or harm are not unethical” [82]. In addition, in the context of ethnocultural competence, the APA recognises that implementing a new “guideline may require an epistemological and power shift in which psychologists acknowledge that local Indigenous/ethnocultural epistemologies and systems of healing are viable approaches through which to address the mental health and wellness of individuals and communities” ([79] p. 25). It appears psychology as a discipline may need to consider CM, similar to psychiatry and medicine, in the context of psychologist and consumer demand. Further, Australian psychology professional associations may need to follow the lead of other international psychology professional associations in terms of their engagement with CM. It will be important for psychology associations to address the risks highlighted above and to develop resources and guidelines that assist psychologists to navigate CM with their clinical practice.

### Strengths and limitations

Our novel, in-depth analysis of current Australian policy is not without limitations. It was not within the scope of this paper to search within policy and guideline documents using an exhaustive list of CM related professions and practices. As such the results of the searches are limited by the specific CM search terms as described in the method. A further limitation of this analysis is only a selection of English-speaking psychology professional association and their websites were included.

## Conclusion

CM approaches are used by consumers to address mental health symptoms and some psychologists are engaging with their clients CM use. However, the current paper highlights there is limited support, in the form of policy and guidelines from the discipline of psychology, for psychologists to engage with CM their clinical practice. There are risks associated with a lack of CM relevant policy and guidelines for psychologists, including disconnection from certain clients and ethnocultural groups who may have a preference for CM treatment options. More research is needed to establish why Australian psychology professional associations have not addressed client preference for, and psychologist interest in, CM as part of mental health treatment. Further research may assist an understanding of how psychologists engage with CM and how psychology can support this engagement through the development of CM relevant policy, guidelines, and education. 

## Data Availability

Data sharing is not applicable to this article as no datasets were generated or analysed during the current study. This study data can be available upon reasonable request to the corresponding author. The authors confirm that the data supporting the findings of this study are available within the article.

## Abbreviations

AAPI: Australian Association of Psychologists Incorporated
ACPA: Australian Clinical Psychology Association
AHPRA: Australian Health Practitioner Regulation Agency
AIPA: Australian Indigenous Psychologists Association
AMPS: Australian Music and Psychology Society
APA: The America Psychological Association
APAC: Australian Psychology Accreditation Council
APACS: Australian Psychologists and Counsellors in Schools
APS: Australian Psychological Society
BPS: British Psychological Society
CAM: Complementary and alternative medicine
CM: Complementary Medicine
CPA: Canadian Psychological Association
PsyBA: Psychology Board of Australia
RANZCP: Royal Australian New Zealand College of Psychiatrists
READ: Ready materials, Extract data, Analyse data, Distil
WHO: World Health Organisation
